# Human Cerebrospinal Fluid Modulates Pathways Promoting Glioblastoma Malignancy

**DOI:** 10.3389/fonc.2021.624145

**Published:** 2021-03-04

**Authors:** Anna Carrano, Natanael Zarco, Jordan Phillipps, Montserrat Lara-Velazquez, Paola Suarez-Meade, Emily S. Norton, Kaisorn L. Chaichana, Alfredo Quiñones-Hinojosa, Yan W. Asmann, Hugo Guerrero-Cázares

**Affiliations:** ^1^Department of Neurological Surgery, Mayo Clinic, Jacksonville, FL, United States; ^2^Neuroscience Graduate Program, Mayo Clinic Graduate School of Biochemical Sciences, Mayo Clinic, Jacksonville, FL, United States; ^3^Regenerative Sciences Training Program, Center for Regenerative Medicine, Mayo Clinic, Jacksonville, FL, United States; ^4^Division of Biomedical Statistics and Informatics, Department of Health Sciences Research, Mayo Clinic, Jacksonville, FL, United States

**Keywords:** glioblastoma, cerebrospinal fluid, cancer progression, tumor stem cells, brain tumor, subventricular zone

## Abstract

Glioblastoma (GBM) is the most common and devastating primary cancer of the central nervous system in adults. High grade gliomas are able to modify and respond to the brain microenvironment. When GBM tumors infiltrate the Subventricular zone (SVZ) they have a more aggressive clinical presentation than SVZ-distal tumors. We suggest that cerebrospinal fluid (CSF) contact contributes to enhance GBM malignant characteristics in these tumors. We evaluated the impact of human CSF on GBM, performing a transcriptome analysis on human primary GBM cells exposed to CSF to measure changes in gene expression profile and their clinical relevance on disease outcome. In addition we evaluated the proliferation and migration changes of CSF-exposed GBM cells *in vitro* and *in vivo*. CSF induced transcriptomic changes in pathways promoting cell malignancy, such as apoptosis, survival, cell motility, angiogenesis, inflammation, and glucose metabolism. A genetic signature extracted from the identified transcriptional changes in response to CSF proved to be predictive of GBM patient survival using the TCGA database. Furthermore, CSF induced an increase in viability, proliferation rate, and self-renewing capacity, as well as the migratory capabilities of GBM cells *in vitro*. *In vivo*, GBM cells co-injected with human CSF generated larger and more proliferative tumors compared to controls. Taken together, these results provide direct evidence that CSF is a key player in determining tumor growth and invasion through the activation of complex gene expression patterns characteristic of a malignant phenotype. These findings have diagnostic and therapeutic implications for GBM patients. The changes induced by CSF contact might play a role in the increased malignancy of SVZ-proximal GBM.

## Introduction

Glioblastoma (GBM) is the most common and aggressive type of primary brain cancer in adults, accounting for 54% of new gliomas and 45% of primary malignant tumors (1). In the United States alone, 15,000 people die and 26,000 new cases are detected annually (2) with an estimated economic burden exceeding US$300 million per year (3). Survival expectancy of a patient suffering from GBM averages 14 months, despite the most advanced therapeutic strategies combining surgery, chemotherapy, and radiation (4, 5). Invariably, GBM will eventually reoccur in almost 100% of cases, due to the highly invasive nature of the tumor that makes complete resection impossible and the presence of a subpopulation of cells called brain tumor initiating cells (BTICs) (6). These cells exhibit neural stem cell (NSC) properties, such as self-renewal and the ability to differentiate into defined progenies (7, 8). BTICs are also more resistant to chemo- and radio- therapy, and if not completely removed during surgical resection, have the capacity to generate new tumors (9).

Tumor location greatly influences the prognosis of GBM patients. More than half of all patients with GBM have tumors that touch the lateral ventricle or even reach into an important brain neurogenic region known as the subventricular zone (SVZ) (10–15). These patients have significantly worse outcomes in terms of median overall survival, time to progression, and recurrence (3, 12).

The cause for worse outcome for patients suffering from SVZ-infiltrating GBMs is not known. Although it is tempting to speculate that the neurogenic characteristics of the SVZ are the underlying causes of this clinically observed phenomenon. The SVZ is the largest neurogenic niche in adults and is highly regulated by the flow of cerebrospinal fluid (CSF). The CSF milieu has the properties to sustain the neurogenic niche environment, regulating neural stem cells proliferation, differentiation, and migration (13, 16–18). Given the neurogenic potential that CSF holds on NSCs and the similarities between NSCs and BTICs, we explored the possibility that CSF might similarly affect GBM proliferation, differentiation, and migration.

The role of CSF in modulating the aggressiveness of GBM, or other gliomas for that matter, is largely understudied in the field of neuro-oncology. In this work we have taken on the hypothesis that CSF contact enhances GBM malignant characteristics and we have resolved to study the effects of CSF exposure on human GBM-derived BTICs. Our study evaluated CSF impact on GBM gene expression profile as well as cell proliferation and migration *in vitro* and *in vivo*. We observed that CSF is an important contributor to tumor growth and invasion through the activation of gene expression patterns characteristic of a malignant phenotype.

## Materials and Methods

### Primary-Cultured BTICs and Human CSF Collection

Under Mayo Clinic institutionally-approved protocol we established primary BTIC cultures from tumor tissue from patients undergoing surgical resection for newly diagnosed GBM without prior treatment as described previously by our group (4, 19). Clinical data for primary BTICs and CSF samples used in this study are described in detail in Supplementary Table 1.

Primary-cultured BTICs derivation and culture protocols were performed as previously described (20, 21). Briefly, intra-operative brain tissue was chemically and mechanically dissociated in Accutase® and cell number and viability were determined by trypan blue exclusion. Cells were maintained in media composed by DMEM/F12 (Invitrogen) supplemented with 2% GEM21 Neuroplex (Invitrogen), 1% antibiotic/antimycotic (Invitrogen), 20 ng/mL human EGF (Peprotech) and 20 ng/mL FGF (Peprotech). Cells were maintained in suspension to evaluate their neurosphere formation and pluripotency *in vitro*. Tumor initiation potential was determined *in vivo* by orthotopic implantation in immunosuppressed mice (J:Nu, Jackson Labs).

For CSF collection and processing, samples were obtained intra-operatively upon opening of the dura mater. CSF samples were spun at 200 × g for 5 min at 4°C, filtered by 0.45 μm to eliminate cells and debris, and immediately aliquoted and stored at −80°C until use.

For CSF stimulation, BTICs were cultured on laminin-coated plates. Twenty-four hours prior to experimentation, cells were maintained in base media (without EGF and FGF) to avoid confounding effects. Protein concentration in CSF was measured and CSF was utilized at a 1:100 dilution in base media, unless otherwise stated. CSF samples were matched based on gender and age, specifically cCSF73 was matched to ncCSF12 or ncCSF1276 and cCSF-37 to ncCSF25. Cells were maintained under CSF stimulation in incubation conditions for 24 h or as required for analysis.

### RNA Expression Microarray

Cells were grown in 25 cm^2^ flasks until 80% confluency was reached, then treated with cancer or non-cancer CSF (CSF was pooled from 3 cases each). Cells were incubated in CSF or control conditions for 24 hrs. Upon incubation, cells were centrifuged and RNA was extracted. Samples were assessed for RNA quality and quantity using a 2100 Bioanalyzer (Agilent) with a RNA 6000 Nano Chip (Agilent) and diluted to a final concentration of 50 ng/μl. All samples used in this study had RIN values >9.2 μg of RNA were submitted for microarray using the Illumina Human HT-12 v4 chip. The scanned images of the microarrays (.dat files) were processed using Illumina's GenomeStudio software. The probe level intensities were quantile normalized across samples with background subtraction. Probes with detection *p*-values > 0.05 in all samples were excluded. All samples had >40% probes expressed and passed this step of QC. In addition, all samples passed the Illumina's internal QC threshold of signal intensity ratios between 5′ vs. 3′ probes targeting house-keeping genes. The bimodal distributions of the normalized and log2 transformed probe intensities were plotted to determine the threshold of expression as 6.6 (data not shown). Genes with average expression values in all experimental groups below the threshold of expression were filtered out. The principle component analyses (PCA) as well as the unsupervised hierarchical clustering analyses of all samples using the remaining 21,023 probes identified two outlier samples which were consequently removed for the following analysis of variance (ANOVA) and weighted gene co-expression network analysis (WGCNA). Source of variance analyses identified CSF treatment, cell line, and CSF treatment/patient interactions as main contributors to variance, and were included in the ANOVA model. Pair-wise group comparisons were also performed to obtain fold change and *p*-values between any two groups.

### Weighted Gene Co-expression Network Analysis and Pathway Analysis

WGCNA (22) was performed to identify modules of highly correlated genes. Briefly, the gene co-expressions were calculated using Pearson Correlated Coefficient to the power of 6 (β = 6) optimized for discoveries of scale-free topology. A signed hybrid Topological Overlap Measures (TOM) matrix were calculated to reduce sporadical correlations between gene-pairs. In addition, modules whose eigengenes highly correlated with treatment groups were tested for enrichment of known functional gene sets using Broad's Molecular Signatures Database (http://software.broadinstitute.org/gsea/msigdb).

Gene ontology (GO) and pathway analysis were performed on differential gene expression (DEG) results using Metacore (Clarivate Analytics) and Ingenuity Pathway Analysis (IPA, Qiagen). DEGs were defined as genes with a Fold change (FC) higher than 1.2 or lower than −1.2 and adjusted *p*-value < 0.05. Canonical pathways were assessed for significant enrichment and directionality of effect utilizing a z score >±2 and *p*-value < 0.05 (right-tailed Fisher's exact-test) on IPA. Network Analysis was also performed on Metacore, using Analyze Networks (AN) algorithm (default settings) to generate a list of biological sub-networks highly enriched and unique for the uploaded data. In this workflow the networks are prioritized based on the number of fragments of canonical pathways on the network, ranked by *p*-value, G-score and interpreted in GO terms. Transcriptional regulators of DEG genes were also identified with MetaCore Interactome. Transcription factors (TF) are ranked according to their *Z*-score (the level of connectivity of the TF to the DEG list). Larger *Z*-scores represent higher levels of connectivity between the transcription factor and the DEG list.

### Molecular and Clinical Data Collection and Patients Survival Analysis

Pre-processed and normalized gene expression data was retrieved from GlioVis portal (http://gliovis.bioinfo.cnio.es/) (mRNA expression from the Affimetrix HT Human Genome U133 array). Unsupervised hierarchical clustering of both genes and samples was performed using Partek Genomics Suite (https://www.partek.com/partek-genomics-suite/, St. Louis, Missouri). The quantile normalized and log2 transformed expression values were used for unsupervised hierarchical clustering of both samples and probes. Pearson Correlation Coefficient was used as the distance metric between samples and genes. The distance between two clusters is defined as the average of distances between all pairs of objects using the average linkage method. The expression values of each gene across samples were standardized to mean of 0 and scaled to standard deviation of 1. Clinical data collection for overall survival and disease/progression free was obtained from the GBM provisional cohort in the cBioPortal for Cancer genomics (https://www.cbioportal.org).

### Cell Viability and Migration Assay

Cell culture growth and viability was evaluated by alamarBlue (Invitrogen). GBM cells were seeded at 2.000 cells/well in a laminin-coated 96-well plate (*n* > 4 per condition) and incubated overnight to allow for attachment in base media. A 10 μL aliquot of alamarBlue reagent was added to the wells containing 100 μL of base growth media with or without treatments, and cells were incubated for at least 4 h at 37°C. Fluorescence was measured using a plate reader (Ex 540–570 nm, Em 580–610 nm) at 24, 48, 72 h after CSF stimulation.

Cell migration response to CSF stimulation was evaluated by transwell and gradient migration assays. For transwell migration, we utilized a modified Boyden chamber. Fifty thousand cells were seeded in culture inserts with an 8 μm pored permeable membrane in 3 replicates per condition, to allow migration to the bottom compartment. CSF was applied to the bottom compartment. Chambers were maintained for 24 h in incubator conditions. Non-migrated cells were removed from the upper compartment and migrated cells were stained with DAPI, and counted at 10X magnification from nine different fields by an independent observer.

For gradient migration assay, cells were plated on a glass bottom multiwell plate (ibidi™) coated with poly-L-ornithine solution (0.01%, Sigma-Aldrich) and laminin. A 2 × 10^5^ cells/ml suspension was plated per chamber and allowed to adhere overnight in base media. CSF was applied on one side of the chamber to establish a CSF gradient. Time-lapse of cell migration was recorded using an inverted microscope with environmental chamber. 10x images were acquired every 10 min for 24 h and processed with Zeiss ZEN Blue software. At least 3 time-lapse videos were collected per conditions. Cells were tracked (at least 30 cells/video) using ImagePro Software (Media Cybernetics).

### *In vitro* Extreme Limiting Dilution Assay

The assay was performed as previously described (23). Briefly, cells were seeded at 1, 5, 10, or 50 cells/well in a laminin-coated 96-well plate and incubated overnight to allow for attachment in base media. The following day, attached cells were counted and treated with CSF. Sphere formation was monitored over a period of 14 days and each well was quantified and scored thereafter. Colonies measuring 250 μm or above were counted as positive. A semilogarithmic plot was generated of the fraction of negative cultures also referred to as “non-responding” (i.e., wells lacking spheres) as a function of the dose of cells placed in each culture. Results were analyzed using the online software tool at the following website from Walter and Eliza Hall Institute of Medical Research: http://bioinf.wehi.edu.au/software/elda/.

### Cell Proliferation

Nuclear Ki67 expression was evaluated to determine cells in active proliferative phases. Cells were fixed in 4% paraformaldehyde in phosphate-buffered saline (PBS) for 30 min, and blocked for 1 h in PBS containing 0.1% Triton X-100 (Sigma-Aldrich) with 10% goat serum prior to overnight incubation with anti-Ki67 (RM-9106-s1; Thermo Fischer Scientific, 1:500). Alexa 594-labeled secondary antibody (Invitrogen, 1:500) was used for visualization, and DAPI was used to counterstain cell nuclei. Slides were visualized and recorded with an inverted fluorescence microscope and the number of Ki67+/DAPI cells was counted in at least 8 randomly selected fields at 20x per slide. For mouse tissue, after fixation in 4% paraformaldehyde and paraffin embedding, brains were sliced at 5 μm, deparaffinazed and treated for antigen retrieval in citrate buffer for 30 min, followed by primary antibody staining as described above.

Cyclin D1 expression was measured by real-time quantitative PCR, after 24 h exposure to CSF. Total RNA was isolated using TRIzol (Invitrogen) and the RNeasy mini kit (Qiagen) following the manufacturer's instructions. Reverse transcription was performed using Superscript III (Invitrogen) and real time quantitative PCR was performed on a Quant Studio 3 (Applied Biosystems) with Power SYBR Green PCR Master Mix. Relative quantification of mRNA expression was calculated by the ΔΔCT method after adjusting the levels to the corresponding internal GAPDH control for each sample. Primers sequences were as follows: human GAPDH sense: 5′-AGGTCGGTGTGAACGGATTTG-3′, antisense 5′-TGTAGACCTGTAGTTGAGGTCA-3′; human Cyclin D1 sense 5′-CAATGACCCCGCACGATTTC-3′, antisense 5′-CATGGAGGGCGGATTGGAA-3′.

### Orthotopic Tumor Implantation

All animal procedures were approved by the Mayo Clinic Institutional Animal Care and Use Committee in accordance with National Institutes of Health guidelines. Athymic nude mice (J:Nu, Jackson Laboratories) were housed three to five per cage and maintained on *ad libitum* access to food and water with a 12 h light/dark cycle. All animals were used for study between 8 and 15 weeks of age.

Glioblastoma cells GBM1A (previously known as GBM 0913) originally established by Vescovi et al. (7) and previously characterized by our collaborators were used for the *in vivo* studies (7, 24–26). To identify GBM cells in our *in vivo* mouse experiments, we transduced the cells with lentiviral vectors coding for Green Fluorescence protein/Luciferase (GFP-Luc) proteins (RediFect™ Red-FLuc-GFP, Perkin Elmer cat: CLS960003). One week post-transduction, cells were sorted by flow cytometry to select GFP-expressing cells. Cells were washed in PBS and resuspended in PBS before injection to remove any trace of growing media.

We performed intracranial injection as described previously by our group (18, 19, 27). Mice were anesthetized using isoflurane and immobilized in a stereotaxic apparatus. 1 × 10^5^ GBM1A GFP-Luc cells were resuspended in 3 μl of CSF (or PBS for controls) plus 2 μl 0.2% Pura Matrix (Corning) and injected intracranially into the right hemisphere (coordinates from bregma in mm: Y: 0.86; X: 2; and Z: −3). Injection was performed with an automatic system at a 0.5 μl/min rate and injection needle was left in place for an extra minute to reduce backflow. Injection needle was then retrieved and skin incision was closed with surgical glue. Isoflurane was discontinued, and the animal placed on a heating pad. All mice completely recovered within 5 min. Buprenorphine (10 mg/kg) was used as pre- and post-operative analgesic.

Mice were randomized into 3 experimental groups of 8 animals each: (1) control GBM1A coinjected with PBS and Pura Matrix, (2) GBM1A coinjected with cancer CSF and Pura Matrix, and (3) GBM1A coinjected with non-cancer CSF and Pura Matrix. Tumor formation and growth were followed by bioluminescence (BLI) every week. Mice were euthanized 4 weeks after injection. Brains were fixed using transcardiac perfusion, and post-fixed overnight at 4°C in 4% paraformaldehyde.

Tumor volume was calculated performing a morphometric analysis of brain sections that presented tumor. The morphometric volume (MFV) determination was done using the Cavalieri principle (28), which allows an accurate estimation of the volume of a structure independently of its shape and size, estimating the surface area (*A*) of a number (*n*) of parallel sections spaced at a constant distance (*t*), using the following equation: *est (V)* = *t* ^*^ *(A1* + *A2* + *A3* +*& An)*. Three 5 μm brain slides per tumor sectioned at 50 μm intervals were used for this calculation.

### Bioluminescence Imaging

*In vivo* bioluminescence images of tumor-implanted mice were obtained using the IVIS Spectrum System (Perkin Elmer) that has a cooled CCD camera to capture images of animals and tissues in a light-tight box. D-luciferin (XenoLight D-Luciferin—K+ Salt Bioluminescent Substrate 15 mg/ml, Perkin Elmer) was injected intraperitoneally at a dose of 10 mg/kg and allowed to distribute for 5 min. Mice were then anesthetized using isoflurane and imaged in prone position. Imaging times ranged from 5 s to 5 min, depending on the total tumor burden as a function of light emission from tumor cells. Region of interest analysis was performed using Living Image Software.The mean ± SD light emission over the time was plotted for each brain tumor engrafted mice. Bioluminescent signal was normalized and analyzed as the absolute total flux (photons/steradian/cm^2^). Mice were imaged at 1, 2, and 3 weeks after tumor engrafting.

### Statistical Analysis

Statistical analysis was performed using GraphPad Prism 8. Multiple comparisons analysis was determined by a one-way ANOVA, followed by Tukey *post-hoc* analysis. Results represent the mean ± SEM of four replicates in three independent experiments unless stated otherwise. Statistical significance is represented by *p < 0.05, **p < 0.01, ***p < 0.001.

## Results

### Pathways Analysis Indicates That CSF Modulates Aggressive Features of GBM

**Figure 1:**
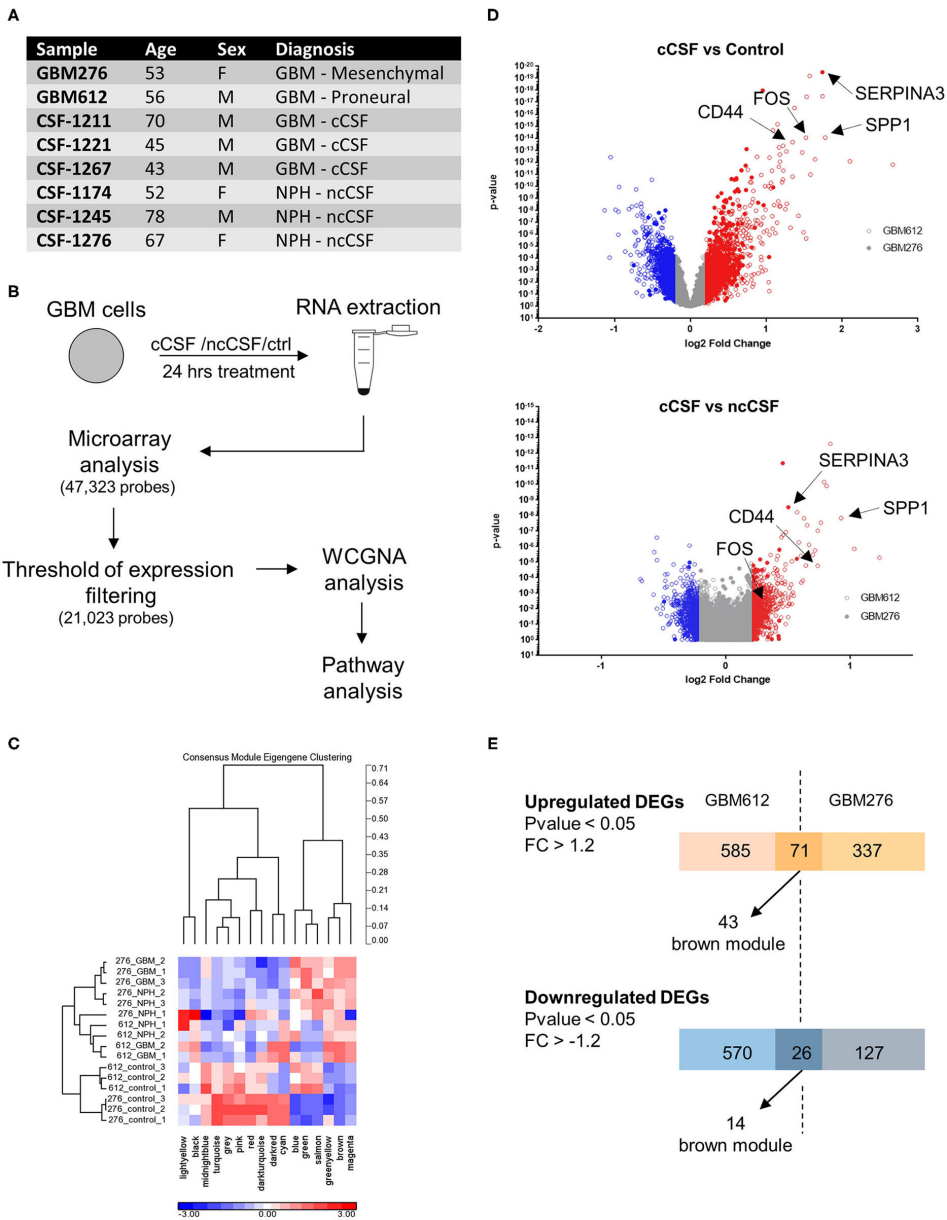
RNA expression microarray WGCNA analysis. **(A)** Demographic data of samples utilized for RNA expression analysis. **(B)** Schematic of the experiment. Cells were cultured for 24 h in the presence of cCSF or ncCSF then processed for RNA extraction and microarray analysis. **(C)** With a Weighted Gene Co-expression Network Analysis (WCGNA) we identify 16 modules of highly correlated genes, of which 10 reached significancy. **(D)** Volcano plot showing the expression levels of the transcripts derived from the 2 cell lines analyzed. GBM276 is represented by diamonds, GBM612 is represented by circles. **(E)** Venn diagrams showing unique and common DEGs between the 2 BTICs cells used for analysis (GBM612 and GBM276). GBM, Glioblastoma; cCSF, cancer derived CSF; ncCSF, non-cancer derived CSF; NPH, normal pressure Hydrocephalus; M, male; F, female.

In order to establish the extend of the effects of CSF contact on GBM, we performed a transcriptome analysis in CSF-treated GBM cells. To the best of our knowledge, there is not data to date on CSF induced transcriptomic changes in GBM cells. To identify common properties of CSF derived from brain cancer compared with non-cancer patients, we have pooled in this study CSF derived from 3 brain cancer patients (high grade gliomas) and CSF from 3 control patients (hydrocephalus) (Figure 1A).

**Figure 2:**
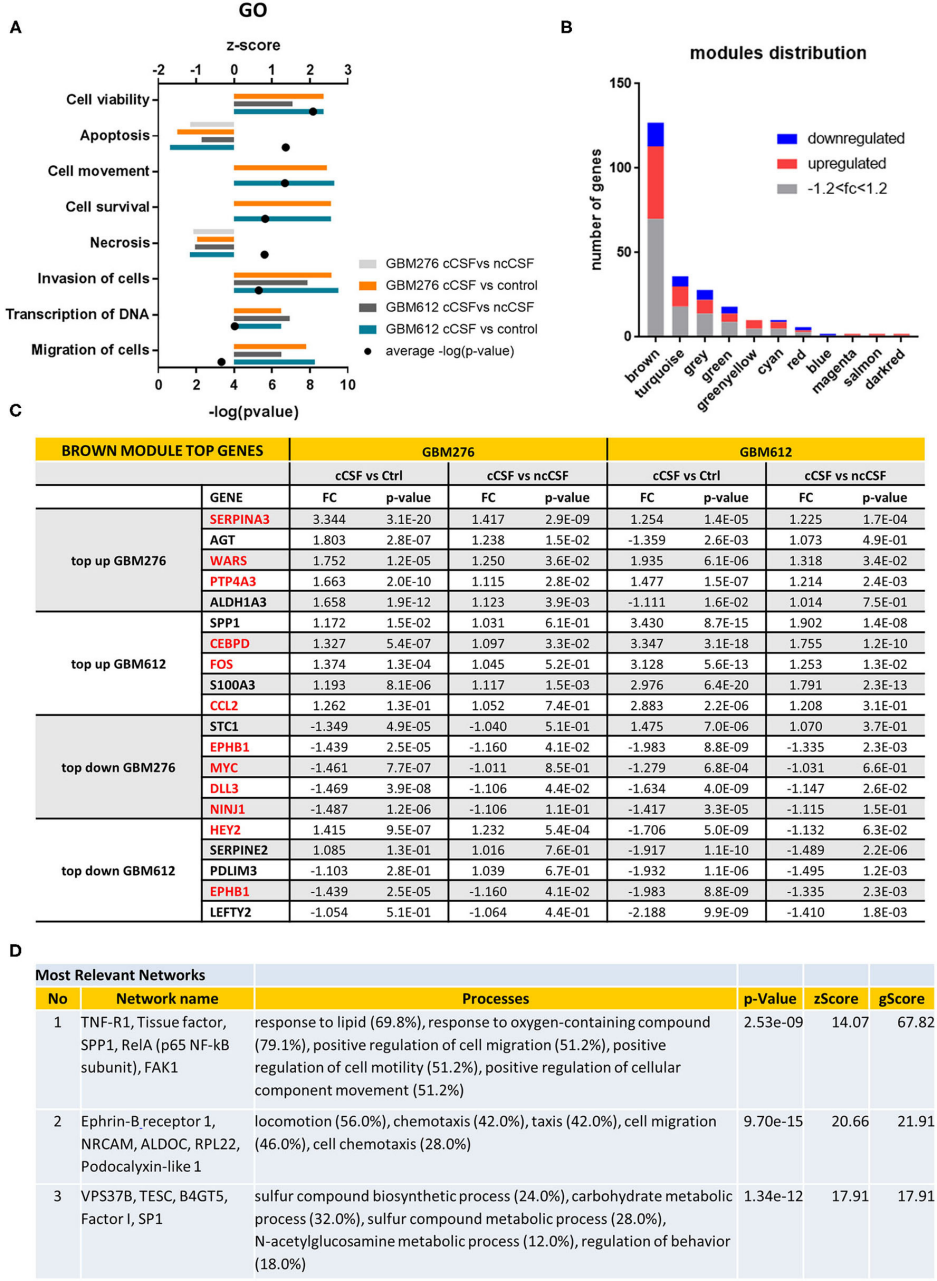
Pathway Analysis of DEGs. **(A)** Top gene ontology processes for DEGs common to both lines tested. Bars represent z-scores for each pairwise comparison, dots represent –log(*p*-value) averages of the 4 pairwise comparisons. **(B)** Distribution of DEGS in WGCNA modules, with the brown module containing most of the significant DEGs defined by *p*-value < 0.05. Red indicates upregulation of fold change +1.2, blue indicates downregulation of fold change −1.2. **(C)** Top 5 up or down-regulated DEGs in brown module for GBM276 and GBM612 with relative fold change (FC) and *p*-value in each pairwise comparison. Common DEGs are represented in red. **(D)** Most relevant networks for common DEGs in the brown module using Metacore algorithm Analyze Networks. FC, fold change; FDR, false discovery rate.

Of the 47,323 probes initially detected, only 21,023 probes remained after filtering and these were used for differential expression analysis (Figure 1B). Consensus module Eigengene clustering confirmed that cancer CSF and non-cancer CSF exposure induces the segregation of GBM samples into intrinsically different subsets, with the control group naturally being the most distant cluster (Figure 1C). Pair-wise group comparisons were also performed to obtain fold change and *p*-values between any two groups. We then analyzed the expression fold change of the GBM-CSF treated samples compared to controls (using a threshold of fold change ±1.2 and *p*-value <0.05), and we found 1,252 differentially expressed genes (DEGs) in the GBM612 sample (of which 656 upregulated, 596 downregulated) and 561 DEGS in the GBM276 sample (of which 408 upreguated, 153 downregulated) (Figure 1D), among these, 97 DEGs were shared between the 2 different GBM primary cultures (71 upregulated, 26 downregulated) (Figure 1E). Most importantly, when we performed a gene ontology (GO) analysis on the common DEGs for pathway enrichment we observed processes involved in regulation of cell invasion, apoptosis, survival, and transcription of DNA (average *p* < 0.001) (Figure 2A), all processes that support the clinical observations of SVZ-proximal GBM being more malignant than the distal counterpart.

### WGCNA Analysis Reveals the Presence of a Biologically Significant Module for GBM Progression

Global analysis of the entire dataset by WGCNA highlighted the relevance of one module (brown module, 828 genes) containing the highest number of differentially expressed transcripts common to both cell lines (Figures 1E, 2B). WGCNA modules group genes based on similar expression profiles that are highly connected and these genes often also share similar biological functions. The brown module contained 127 common DEGs, of which 43 upregulated and 14 downregulated by cancer CSF exposure in both cell lines. The top 5 upregulated genes in the brown module for GBM276 and GBM 612 were SERPINA3, AGT, WARS, PTP4A3, ALDH1A3, and SPP1, CEBPD, FOS, S100A3, CCL2 respectively. The top 5 downregulated genes in the brown module for GBM276 and GBM 612 were respectively NINJ1, DLL3, MYC, EPHB1, STC1, and LEFTY2, EPHB1, PDLIM3, SERPINE2, HEY2 (Figure 2C and Supplementary Figure 1A). The enrichment analysis of the entire brown module in Metacore revealed GO and process networks terms involved in angiogenesis, cell adhesion and integrin signaling, cell proliferation and response to inflammatory stimuli, again suggesting the role of CSF in promoting GBM aggressive characteristics (Figure 2A and Supplementary Figures 1B,C) The unique genes from the top 15 pathways in the brown module are listed in Supplementary Table 2. Using the Analyze network algorithm in Metacore, we have identified the most connected items in the brown module collected in 3 main networks as shown in Figure 2D. These biological networks again confirmed the positive regulation of cell migration by CSF, as well as metabolic and biosynthetic processes which are also affected during tumorigenesis. Upstream regulator analysis using MetaCore showed key transcription factors whose targets are overrepresented in the differentially expressed gene lists, that are involved in the regulation of the pathways mentioned above, such as MYC, STAT3, and FOS, which were also found upregulated in our dataset (Supplementary Table 3).

The results of this analysis summarize the complexity of functions regulated by the CSF in the contest of GBM pathophysiology.

### CSF-Induced Gene Expression Signature Determines Patients' Clusters With Different Survival Patterns

**Figure 3:**
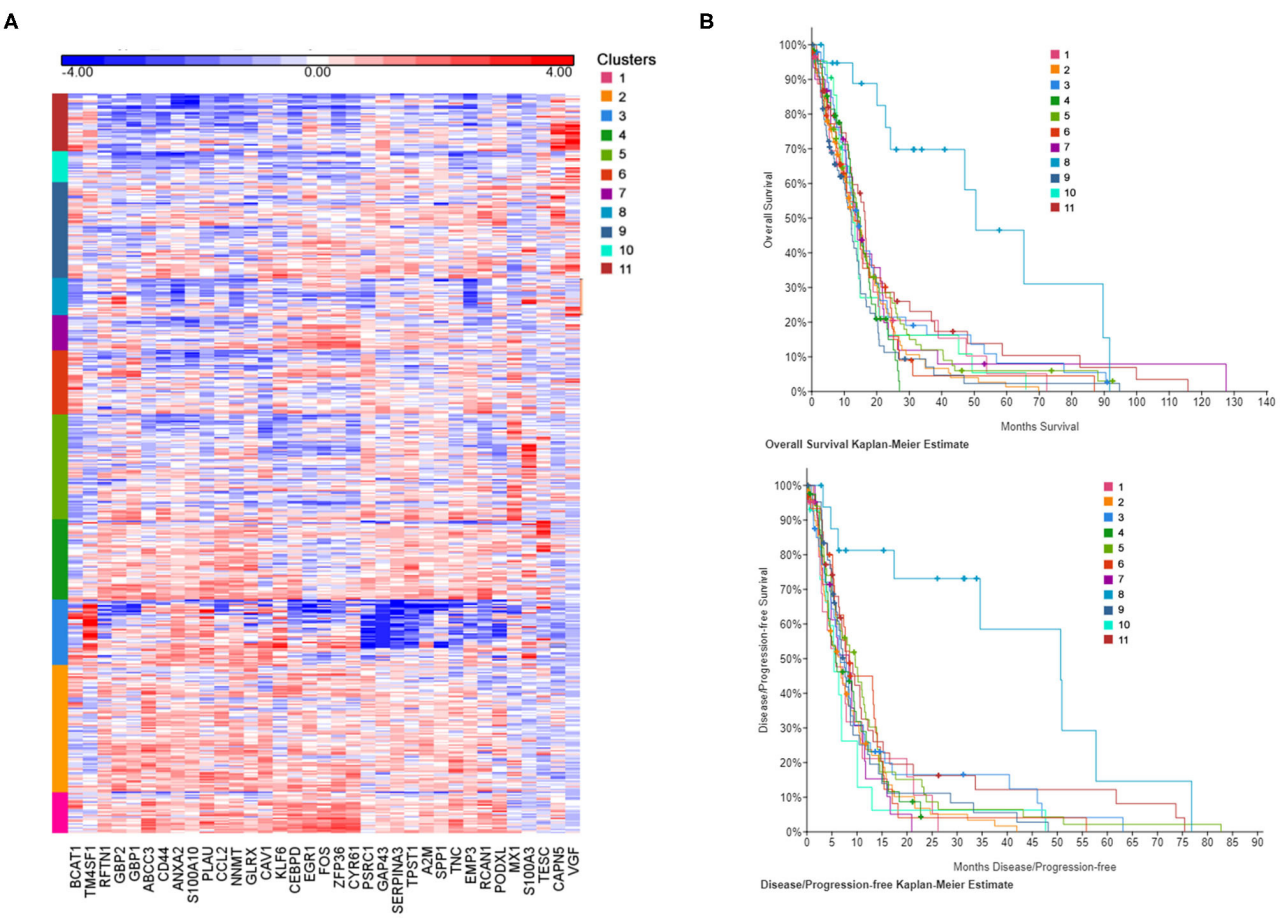
CSF-induced gene signature influence survival in GBM patients. **(A)** The heat map represents each expression component. Each row represents a patient sample and each column a gene selected for a CSF-induced signature in GBM *in vitro*. For each patient, red indicates upregulation, blue indicates downregulation. Clusters (1–11) are color coded and represent gene-specific expression patterns. **(B)** Overall survival and disease/progression free Kaplan-Meier plots for the clusters derived by CSF-induced gene expression show a distinct cluster (8) with significant better clinical features.

In order to establish the clinical relevance of the gene signature induced by CSF in GBM cells *in vitro*, we have attempted to identify patient subgroups in the TCGA database that carry this particular gene expression profile. We have selected DEGs from Figure 1D (cCSF vs. control analysis) with a *p*-value < 0.05 and a FC > 2 in either cell line analyzed and generated a list of 35 upregulated genes to represent the CSF-induced gene signature in GBM. Gene expression measurements for the entire dataset in the TCGA GBM provisional cohort were retrieved from GlioVis portal (http://gliovis.bioinfo.cnio.es/). We then performed independent hierarchical clustering of both genes and patient samples and we generated 11 patient clusters with different transcriptomic profiles (Figure 3A). We next determined whether these clusters in the TCGA data were associated with differences in survival outcomes. Kaplan-Meier (KM) analyses revealed the existence of a cluster (Cluster 8) with significant longer overall survival (OS) and disease/progression free survival (DFS) (Figure 3B). Cluster 8 patients show an overall reduced expression of the 35 up-regulated signature genes, which all in concert contributed to the clinical outcome differences (Supplementary Figure 2). The genes with the most significantly low expression (compared to the TCGA cohort) were EMP (*p* < 0.0001), NNMT (*p* < 0.0001), CCL2 (*p* = 0.002), GBP1 (*p* = 0.0004), RCAN1 (*p* = 0.0044), SERPINA3 (*p* = 0.0075), ZFP36 (*p* = 0.018), CEBPD (*p* = 0.0423), and TNC (*p* = 0.0462).

### CSF Derived From GBM Patients Induces an Increase in BTIC Proliferation

**Figure 4:**
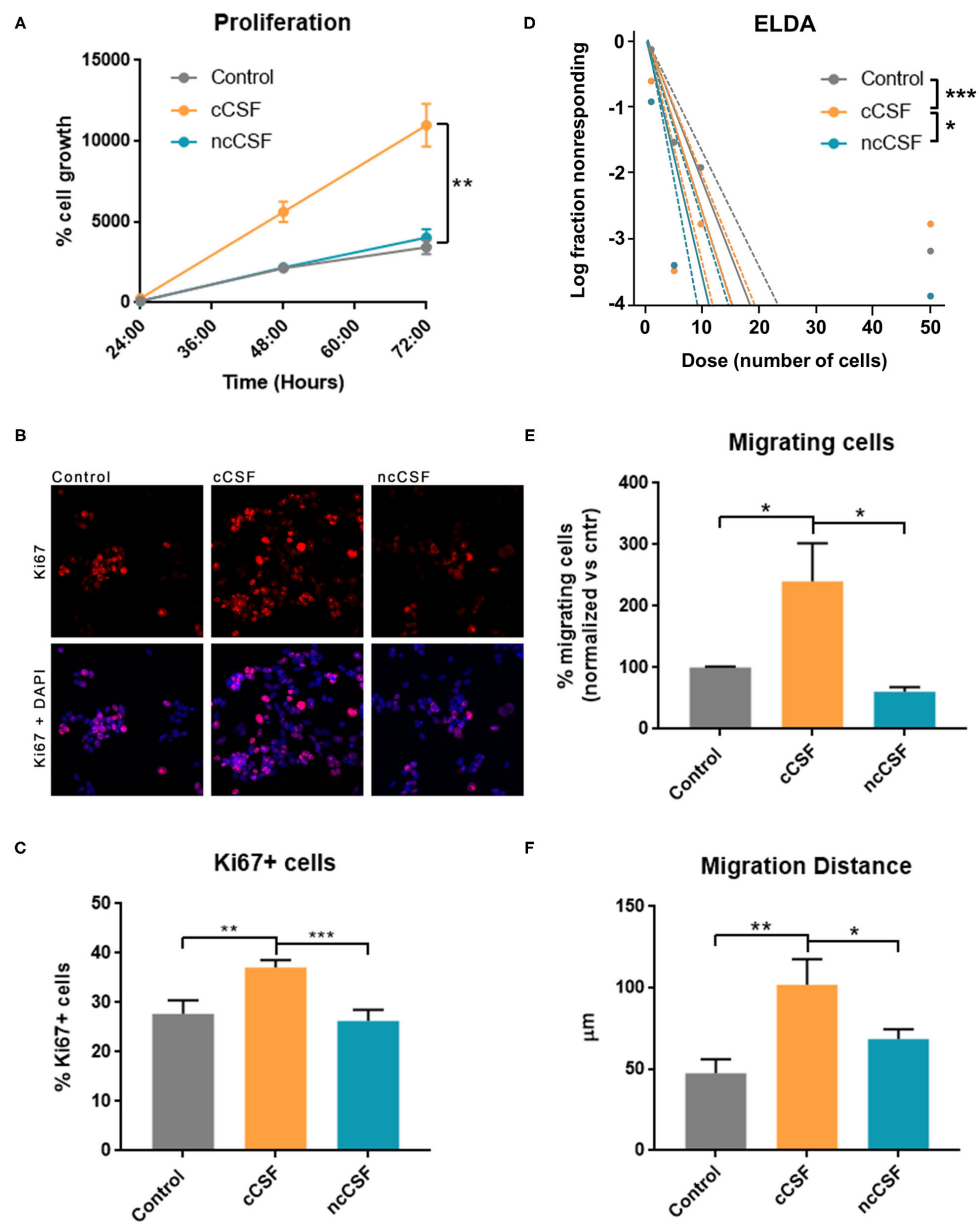
CSF derived from GBM patients induces an increase in BTIC proliferation and migration *in vitro*. GBM cells were treated for 24 h with either glioma derived CSF (cCSF) or non-cancer (ncCSF). After incubation time cells were processed for analysis. **(A)** Alamar blue assay tracked cells proliferation over time (graph shows growth between 24 and 72 h post-treatment) revealing an increase proliferation rate in the cCSF treatment group (CSF pairs: cCSF73/ncCSF12) **(B)** Immunohistochemistry evaluation for proliferation marker Ki67 shows a predominant presence of positive cells in the cCSF treated groups, confirmed by quantification in **(C)** (cCSF on GBM1A: 151.38% increase vs. ncCSF, *p* = 0.0002; 130.33% increase vs. untreated, *p* = 0.0004; cCSF on GBM965: 189.28% increase vs. ncCSF, *p* = 0.01; 104.70% increase vs. untreated, *p* = n.s.) (CSF pairs: cCSF73/ncCSF1276). **(D)** Extreme Limited Dilution Assay (ELDA) determines cells' self-renewal by measuring their ability to form colonies when seeded at a very sparse density. When cells were incubated with cCSF, their propensity to generate colonies was higher compared to the other treatments. *p*-Values: Control vs. ncCSF *p* = 0.175, Control vs. cCSF *p* = 0.000882, cCSF vs. ncCSF *p* = 0.0425 (CSF pairs: cCSF73/ncCSF12). **(E)** Transwell migration assay allows to quantify the number of cell that migrate through a porous membrane. Cells treated with cCSF demonstrated a higher tendency to migrate when compared to untreated and ncCSF. (250% increase cCSF vs. ncCSF, *p* = 0.0429; 157.84% increase cCSF vs. control, *p* = n.s.) (CSF pairs: cCSF37/ncCSF25). **(F)** GBM cell migration in a 2D gradient, cells exposed to cCSF migrated longer distances. (215.24% increase cCSF vs. control *p* = 0.004; 149.04% increase cCSF vs. ncCSF *p* = 0.04, ncCSF vs. control *p* = n.s.) (CSF pairs: cCSF73/ncCSF12). Scale bars = mean ± SEM. *p < 0.05, **p < 0.01, ***p < 0.001. Graphs are representative of gender/age matched pairs of CSF samples.

To validate the results obtained from the pathway analysis, we next evaluated whether CSF is capable of modulating growth, migration, and stem cell features of GBM-derived BTICs. We have assessed proliferation and viability of 2 different BTICs lines by Alamar blue assay upon exposure to human CSF samples (Figure 4A). Both treated cell lines showed significantly different viability in response to the two types of CSF, with the cancer CSF (cCSF) inducing a greater viability rate when compared to non-cancer CSF (ncCSF) and control (*p* < 0.01). This response was proved to be dose-dependent (data not shown). Interestingly, GBM1A and GBM965 showed maximum response at different concentrations, with the GBM1A line being the most susceptible to respond to treatment (Supplementary Figure 3A). No evident morphological differences were observed in GBM cells after exposure to CSF of either origin (Supplementary Figure 3B). Single pairs of cCSF and ncCSF, matched on age and gender, were used for these arrays. When comparing CSF samples from several patients, we consistently observed very mild effects in response to ncCSF in GBM lines, in contrast to cCSF (Supplementary Figure 3C).

The increase in proliferation rate induced by cancer CSF was also substantiated by immunohistochemical staining for Ki67, which labels actively proliferating cells. We observed a significantly higher percentage of cells Ki67 positive compared to the other treatments (*p* < 0.01, Figures 4B,C). We also measured a significant increase in mRNA levels of Cyclin D1, an important cell cycle regulator, required to overcome the restriction point in the G1 stage of the cell cycle in cCSF-treated BTICs compared to controls (*p* < 0.01, Supplementary Figure 3D).

Furthermore, a significant increase in self-renewing capacity was observed in both cell lines following cancer CSF exposure using ELDA, as cCSF treated BTICs produced more readily and bigger colonies than in any other condition (cCSF vs. control *p* = 0.0008, cCSF vs. ncCSF *p* = 0.0425, ncCSF vs. control *p* = n.s.) (Figure 4D). Taken together, our results demonstrate that CSF obtained from GBM patients promotes tumor cell proliferation and self-renewal capacity in GBM cells *in vitro*.

### CSF Derived From GBM Patients Induces an Increase in BTIC Migration

Here we evaluated the cell migration response of BTICs to human CSF by transwell assay and video-microscopy on a 2D surface during 24 h as described previously (17, 29). GBM-derived CSF treated cells showed an increase in their transwell migratory capacity compared to the other treatments (*p* < 0.05, Figure 4E). We also followed cell migration in response to a CSF gradient, in a μSlide IV (IBIDI) for 24 h using timelapse microscopy. This assay confirmed that cCSF enhances migration in GBM cells. Cell migration distance was highest in cCSF-treated BTICs (*p* < 0.05, Figure 4F and Supplementary Figure 4A). Speed was also significantly increased by CSF compared to control (*p* < 0.0001, Supplementary Figures 4B,C). CSF from both sources appeared to have a repellent effect on GBM cells inducing migration in the opposite direction of the gradient (Supplementary Figures 4D,E). These results demonstrate that CSF can enhance the migratory capabilities of GBM-derived BTICs *in vitro*.

### CSF Promotes GBM Growth *in vivo*

**Figure 5:**
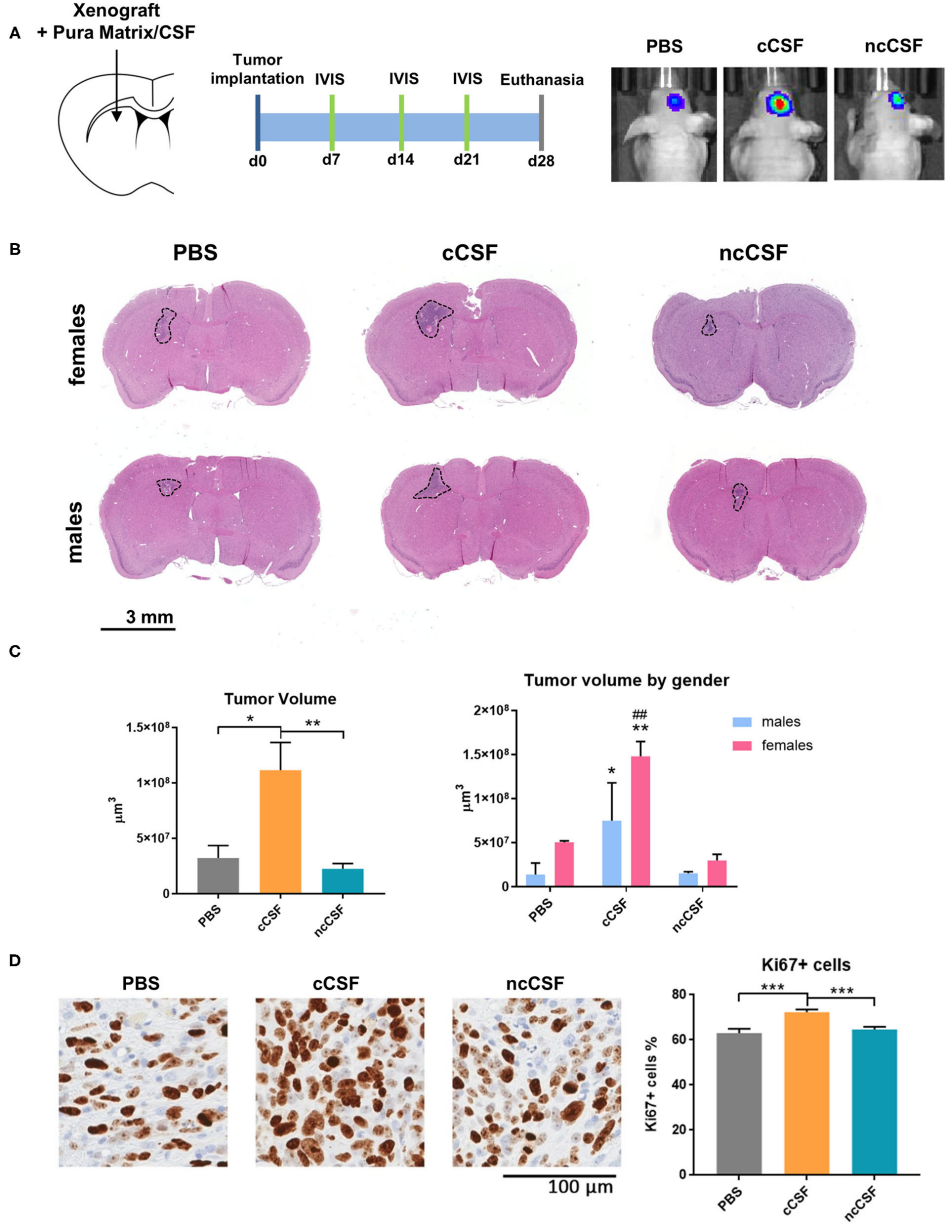
CSF promotes GBM growth *in vivo*. **(A)** Schematic representation of the *in vivo* study design, showing injection site and timeline of the experiment. **(B)** Implanted tumor size. Mice receiving GBM cells co-injected with cCSF harbored larger tumors compared to the other groups. Interestingly, female mice developed bigger tumors than males. **(C)** Quantification of the tumor volumes overall and segregated by female and male mice in the different treatment groups. (*p < 0.05, **p < 0.01 for cCSF vs. PBS; ##*p* < 0.05 for cCSF vs. ncCSF). **(D)** Immunohistochemistry staining for Ki67 in xenograft. A higher percentage of KI67+ cells were observed in the cCSF coinjected cohort. Scale bars = mean ± SEM. *p < 0.05, **p < 0.01, ***p < 0.001.

The effects of CSF, from cancer or non-cancer source, on GBM have never been reported *in vivo*. In order to determine whether the *in vitro* effects of CSF on BTIC proliferation and migration persist when cells are in the brain microenvironment, we tested using a human orthotopic GBM model in mice (30). To evaluate the effect of CSF *in vivo*, we injected GBM cells encapsulated in a CSF-containing hydrogel (Pura Matrix). PuraMatrix is a non-toxic, biodegradable, and biocompatible hydrogel composed by 16 amino acids (RADA16; AcN-RADARADARADARADA-CONH2). This hydrogel mimics several properties of the natural extracellular matrix in which cells can easily proliferate, differentiate, and migrate (31, 32) and, in our case, allowed the creation of a CSF-enriched microenvironment surrounding the implanted BTICs. The use of a hydrogel allowed the CSF to remain in contact with the GBM cells. Importantly, xenographs were generated at a location distal to the SVZ or the subgranular zone. This location allowed us to avoid any interference of the mouse neurogenic niche proximity on our readouts (Figure 5A). A single pair of age/gender matched CSF samples was used for this study (cCSF73/ncCSF1276). Bioluminescence imaging, collected throughout the experiment, demonstrated an increased tumor growth in the group co-injected with cCSF compared to vehicle and CSF from control patients (Supplementary Figure 5A). Upon euthanasia, we observed differences in tumor volume between our groups as identified by H&E staining (Figure 5B) and immunohistochemistry against eGFP (Supplementary Figure 5B). Brain histology showed cCSF co-injected tumors were significantly larger compared to the other 2 groups (cCSF 345.45% larger than PBS, *p* = 0.0169, cCSF 493.12% larger than ncCSF, *p* = 0.0089, ncCSF vs. PBS *p* = n.s). Interestingly, female mice developed larger tumors than their male counterparts (Figure 5C). In addition, immunohistochemistry against Ki67 revealed again a higher proliferative rate in cCSF-coinjected mice (cCSF vs. control *p* < 0.001, cCSF vs. ncCSF *p* < 0.0001, ncCSF vs. control *p* = n.s.) (Figure 5D). No differences were observed between genders in Ki67 positivity. Our results demonstrate that cCSF alone exacerbates the progression of GBM tumors *in vivo*.

## Discussion

In this work we first demonstrated by transcriptomic analysis that CSF induces changes in pathways regulating apoptosis, survival, cell mobility, angiogenesis, response to inflammatory stimuli and metabolism. Based on these results, we identified a brain cancer CSF-induced gene signature linked to survival outcome in GBM patients in the TCGA database. We then further validated our findings *in vitro* and *in vivo* confirming that CSF exposure to GBM-derived BTICs affects their malignant phenotype. Our functional experiments showed that CSF modulates the most critical features of aggressive behavior in cancer cells: proliferation, invasiveness and tumor initiation.

As briefly mentioned earlier, clinical outcome for GBM tumors is strongly influenced by their location in the brain (3, 12). It is thought that tumors arising close to the SVZ (SVZ+) in the lateral ventricles (LV), have a much more aggressive profile than SVZ-distant tumors (SVZ–) (11). SVZ+ GBMs recur at distant locations of the brain with a higher incidence than SVZ-distal tumors and within a shorter period of time (14, 33), this could be related to an increased in cell migration due to the closeness to the CSF as seen in our results. Moreover, studies evaluating survival suggest that patients with SVZ+ tumors tend to have a decreased overall survival and a worse prognosis, which could be related to an increase in cell proliferation and migration combined, due to CSF contact. A study conducted by Chaichana K, et al. in 2008, in which 26 out of 69 patients presented with contrast-enhancing lesions in close contact to the LV, presented a lower median overall survival when compared to patients with SVZ– GBM, 8 vs. 11 months respectively (12). These results were further confirmed by other studies, demonstrating that regardless of tumor size, patient's characteristics and extent of resection, contact to the SVZ represents an independent factor for survival (33, 34).

Altogether, these characteristics support the theory that SVZ+ and SVZ– GBMs carry a different biological behavior, concluding that SVZ+ GBM has a much more aggressive profile with a worse overall prognosis for patients. The prognostic difference could theoretically stem from intrinsic characteristics of the tumor, or from its SVZ-contacting or SVZ-distal location. At this time, there is no conclusive evidence that links the clinical features of SVZ+ GBM to a defined molecular subtype or other intrinsic tumor characteristic (35). Although some have shown a differential distribution of GBM subtype in preferential brain regions (36), a recent work found no differences in molecular signature in tumor bulk tissue from SVZ+ or SVZ– tumors (37). This furthermore suggests that SVZ proximity does not lead tumors to evolve toward a certain molecular subtype or another, nor selects for survival of an intrinsically more aggressive cellular subtype. All of this evidence supports the conclusion that discrepancies in clinical outcomes are not due to intrinsic characteristics of the tumor as a whole, but rather to the environment of its location (38), i.e., the resident SVZ cell population (NPCs) (39) and, importantly, as supported by our results, the CSF and its components, creating a permissive tumor-supportive environment in which any GBM subtype thrives. Studies using directed biopsies in SVZ+ GBM patients and possibly single cell analysis, although often technically very challenging, would be necessary in the future to further dissect the local changes induced by the brain environment.

We have shown here that pathways pivotal for cancer development are indeed modulated by exposure to CSF, particularly when derived from cancer patients (which has obvious implications in the context of tumor recurrence), as regulation of genes involved in cell proliferation, migration, angiogenesis, metabolism and inflammatory responses was observed in our microarray analysis. Most of these pathways converge upstream on the activation of common master regulator genes, such a STAT3, MYC, and FOS which have been proven consistently to be involved in tumorigenesis (40), although have never been studied in the context of or linked to SVZ proximity or CSF exposure.

One of the most significant pathways identified by our analysis involves the upregulation of STAT3 and SERPINA3, which could both be induced by the presence of cytokines of the IL-6 family in the CSF (41). These cytokines are involved in a variety of biological activities such as inflammation, remodeling of extracellular matrix and modulation of cell growth and differentiation via the induction of important regulator elements of these processes, such as C/EBPD, VEGF, Cyclin D1, Matrix metallopeptidase 1 (MMP1) and TIMP metallopeptidase inhibitor 1 (TIMP-1). SERPINA3, not only can be induced by cytokines through STAT3 (42), but itself could be mediating and/or enhancing the same processes as an extracellular soluble protein present in the CSF (43, 44). Converging on the same pathways, we have identified the upregulation of CD44, which may be mediated by the binding of one of its ligands SPP1 (the expression of which is also induced by CSF in our study) and/or MIF (both cytokines present in the CSF), with the coincidental phosphorylation of ERK1/2, c-Jun and c-Fos. This would lead to the induction of a plethora of processes involved in oncogenesis, including proliferation, migration and angiogenesis, all driven by the interaction of GBM cells with CSF. It is possible that the presence of other cytokines in the CSF, highlighting here a crucial role for the inflammatory response, can sustain the overexpression of SERPINA3, among other genes, and together concert the buildup of a tumor supportive environment. Although hypothesizing the identity of such possible modulator candidates is fascinating, it goes beyond the scope of this study; here we have not conducted a systematic analysis of CSF molecules, and we particularly wanted to focus on the effects elicited by CSF, and the downstream targets of this interaction, on GBM cells and GBM cells behavior. The leads generated by this study are presently investigated in our laboratory, we have recently proven in details the role of SERPINA3, one of the top DEGs identified by this analysis, in enhancing GBM tumorigenesis (44).

Consistent with the fact that the SVZ is the largest neurogenic niche in direct contact with the CSF, and with the above clinical features of GBM, our work suggests a pivotal role for CSF in GBM malignancy and progression. As we have shown here, the gene expression changes induced by CSF in GBM cells have important repercussion for the clinical outcome of these patients: the 35 CSF-induced signature genes that we used in our analysis, showed an average lower expression in patients with a significantly better survival. This suggests that modulation of targets of CSF components could contribute significantly to disease outcome. It is crucial for future studies to investigate the nature of the CSF components responsible for the changes observed in GBM cells that we have reported here, and identify the upstream regulators of such tumorigenic effects. Such CSF components could be soluble proteins or ligands present in suspended cells or exosomes. Previous reports have found both populations of shed tumor cells as well as immune cell sets in the CSF of GBM patients. However, it has been described that the lymphocyte population of the CSF in malignant brain tumors has a far fewer percentage of T cells than non-tumoral neurosurgical disorders, implicating depressed cell-mediated immunity of the cells in the CSF (45). Additionally, recent research has identified an exosome-contained protein, LGALS9, in GBM patient CSF that binds to the TIM3 receptor of dendritic cells, inhibiting antigen recognition and presentation. This ultimately leads to failure of the cytotoxic T-cell-mediated anti-tumor immune response (46). Therefore, because the immune cells within the CSF of GBM patients are already immunosuppressed and are unable to respond to tumor antigens, we believe that the response of GBM cells would likely be very similar to the unfiltered CSF as to the filtered CSF used in our study.

An interesting and quite surprising observation in our *in vivo* study was the increased tumor size in female mice compared to males. This is also somewhat in contrast with the clinical observation that GBM is more prevalent and more severe in male patients than females (47). Due to the fact that no significant differences were observed in the proliferation marker Ki67, our results may reveal an ulterior role for either CSF and/or recipient gender in tumor implantation survival rather than solely proliferation. On the other hand, the factors influencing this phenomenon could be various, and not controlled for in this study, among which the original gender of both GBM cells and CSF donors and the gender of the xenographs recipients, and their participation and contingent role in GBM progression should all be further elucidated.

The relatively small number of samples in our study is a limitation due the heterogeneity of GBM and the impact that age, gender, and disease stage have on CSF composition. Future studies with a wider cohort of cases are needed to corroborate our findings and investigate the specific contribution of sex, age, tumor location, and molecular status in the interplay between CSF and GBM progression.

To summarize here we have proven that:

1) CSF alone enhances proliferation and tumor initiating properties of GBM cells;
2) CSF promotes GBM cells migration and invasiveness, regardless of LV proximity;
3) CSF affects GBM cells behavior, *in vitro* and *in vivo* and it alters GBM gene expression to induce a malignant signature with relevant clinical implications.

That CSF modulates GBM is extremely important in explaining what we observe in clinical settings of increased aggressiveness of those tumors close to the SVZ. Components of the CSF, once identified, should be the target of the next class of drugs used to treat this disease.

## Data Availability Statement

The data presented in this study are deposited in the GEO repository, accession number GSE161528.

## Ethics Statement

The studies involving human participants were reviewed and approved by Mayo Clinic Internal Review Board. The patients/participants provided their written informed consent to participate in this study. The animal study was reviewed and approved by Mayo Clinic Institutional Animal Use and Care Committee.

## Author Contributions

AC, KC, AQ-H, YA, and HG-C: conceptualization. AC, NZ, JP, ML-V, PS-M, KC, AQ-H, and HG-C: methodology. AC, NZ, JP, ML-V, PS-M, EN, YA, and HG-C: data analysis. AQ-H, YA, and HG-C: supervision. AQ-H and HG-C: funding acquisition. All authors have reviewed, edited, read, and agreed to the published version of the manuscript.

## Conflict of Interest

The authors declare that the research was conducted in the absence of any commercial or financial relationships that could be construed as a potential conflict of interest.
