# Supplementary material for: Human Cerebrospinal Fluid Modulates Pathways Promoting Glioblastoma Malignancy
Source: Front Oncol. 2021 Mar 4;11:624145. doi: 10.3389/fonc.2021.624145 (PMC7969659; doi:10.3389/fonc.2021.624145)
Supplement: Supplementary file 1 [file Data_Sheet_1.PDF]

SUPPLEMENTARY

Suppl. Table 1

Sample	Age	Sex	Diagnosis	experiments
**Primary BTICs**				
**GBM276**	53	F	GBM - Mesenchymal	microarray
**GBM612**	56	M	GBM - Proneural	microarray, *in vitro*
**GBM965**	61	F	Classical	*in vitro*
**GBM1A**	n/a	M	Classical/Proneural	*in vitro*, *in vivo*
**CSF samples**				
**CSF-12**	57	F	Chiari Decompression - ncCSF	*in vitro*
**CSF-25**	53	M	right temporal lobectomy for epilepsy -ncCSF	*in vitro*
**CSF-37**	62	M	GBM - cCSF	*in vitro*
**CSF-73**	67	F	anaplastic Oligodendroglioma - cCSF	*in vitro, in vivo*
**CSF-1174**	52	F	NPH - ncCSF	microarray
**CSF-1211**	70	M	GBM - cCSF	microarray
**CSF-1221**	45	M	GBM - cCSF	microarray
**CSF-1245**	78	M	NPH - ncCSF	microarray
**CSF-1267**	43	M	GBM - cCSF	microarray
**CSF-1276**	67	F	NPH - ncCSF	*in vitro*, *in vivo*, microarray

Suppl. Table 1

Summary of characteristics of primary BTICs culture and CSF samples used in this study.

GBM (Glioblastoma), cCSF (cancer derived CSF), ncCSF (non cancer derived CSF), NPH (normal pressure Hydrocephalus), M (male), F (female).

SUPPLEMENTARY

Suppl. Table 2

	GBM276				GBM612			
	cCSF vs Ctrl		cCSF vs ncCSF		cCSF vs Ctrl		cCSF vs ncCSF	
Unique genes from top 15 pathways	FC	p-value	FC	p-value	FC	p-value	FC	p-value
A2M	1.38537	2.43E-02	-1.01671	9.03E-01	2.81449	7E-07	1.17274	3.45E-01
AK3	1.13587	2.98E-02	1.05178	3.67E-01	-1.07511	3E-01	1.01274	8.52E-01
AKT3	-1.02261	4.43E-01	1.0176	5.48E-01	1.0448	2E-01	1.01283	7.19E-01
ALDOC	-1.24118	5.76E-05	-1.05381	2.41E-01	-1.56513	5E-09	-1.18702	3.98E-03
Angiotensinogen	1.80271	2.77E-07	1.23812	1.51E-02	-1.35946	3E-03	1.07276	4.87E-01
ARHGEF1 (p115RhoGEF)	1.04125	4.46E-01	1.01725	7.46E-01	1.13731	4E-02	1.0099	8.79E-01
ARHGEF3	1.04125	4.46E-01	1.01725	7.46E-01	1.13731	4E-02	1.0099	8.79E-01
ARPC4	-1.06695	4.11E-01	-1.03295	6.79E-01	1.29028	7E-03	1.02132	8.26E-01
B4GT5	1.22823	2.16E-04	1.17463	2.22E-03	1.36064	6E-06	1.16874	1.20E-02
Bcl-6	1.33809	1.27E-07	1.09812	2.25E-02	1.25358	3E-05	1.14638	7.86E-03
BMP2	1.09806	2.68E-02	1.00449	9.11E-01	-1.16102	3E-03	1.05473	2.82E-01
C/EBPdelta	1.32679	5.39E-07	1.09666	3.31E-02	3.34669	3E-18	1.75456	1.22E-10
Calgizzarin	-1.10008	8.56E-03	1.0089	7.91E-01	1.27211	1E-06	1.28292	3.39E-06
CCL2	1.26211	1.33E-01	1.05181	7.38E-01	2.88262	2E-06	1.20799	3.12E-01
CD44	1.12128	2.53E-02	-1.03087	5.30E-01	2.54654	2E-14	1.50321	5.30E-07
CDK2	1.02198	6.95E-01	1.00104	9.85E-01	1.25376	4E-02	1.13193	2.80E-01
c-Fos	1.3736	1.27E-04	1.04521	5.25E-01	3.12768	6E-13	1.25306	1.33E-02
CHST3	1.28117	7.70E-05	1.08226	1.37E-01	1.21169	3E-03	1.15989	2.71E-02
c-Myc	-1.46104	7.74E-07	-1.01068	8.51E-01	-1.27879	7E-04	-1.03052	6.64E-01
CNTN1 (F3)	1.31698	2.56E-03	1.02641	7.50E-01	1.51877	1E-04	1.31247	1.19E-02
CXCR4	-1.02428	4.75E-01	-1.0771	3.47E-02	-1.03185	4E-01	-1.01072	7.94E-01
DLL1	-1.1765	1.60E-02	1.02067	7.46E-01	-1.37272	2E-04	-1.12274	1.43E-01
DPF3	1.04275	3.19E-01	1.01978	6.38E-01	1.72799	5E-11	1.54665	1.49E-08
Ephrin-A	-1.08439	1.10E-01	1.00099	9.84E-01	1.19493	3E-03	1.06686	2.89E-01
F263	-1.0948	6.45E-02	-1.0509	2.98E-01	1.0079	9E-01	1.01289	8.24E-01
Factor D	-1.06661	1.02E-01	1.07442	7.06E-02	-1.10828	2E-02	-1.00284	9.52E-01
FAK1	-1.06244	8.80E-02	-1.08027	3.29E-02	1.10618	1E-02	-1.0084	8.42E-01
FKHR	1.12156	7.35E-02	1.00318	9.59E-01	-1.25754	3E-03	-1.02229	7.71E-01
GFAP	1.35783	2.23E-04	1.05739	4.30E-01	1.84851	7E-08	1.09075	3.18E-01
HXK2	-1.27463	2.24E-06	-1.02322	5.55E-01	-1.12897	1E-02	-1.06366	2.02E-01
Insulin processed	-1.05655	1.47E-01	1.02912	4.41E-01	-1.09657	3E-02	-1.0129	7.78E-01
JunB	1.30052	3.51E-05	1.00211	9.67E-01	1.54101	1E-07	1.05486	4.00E-01
KAP3	-1.11879	3.54E-02	-1.121	3.26E-02	1.09394	1E-01	-1.01923	7.59E-01
LDHA	-1.08152	9.17E-02	1.0088	8.45E-01	1.55977	6E-08	1.19094	9.15E-03
LPP1	-1.2266	4.18E-03	-1.0524	4.33E-01	-1.15118	6E-02	-1.05545	4.98E-01
LXR-alpha	1.0648	7.72E-02	-1.00552	8.72E-01	1.11504	9E-03	-1.093	4.34E-02
MA1C1	1.53916	1.73E-10	1.16145	8.58E-04	1.44516	2E-08	1.33589	3.95E-06
MAP2	-1.23022	1.08E-04	-1.11462	2.20E-02	-1.0628	2E-01	1.07143	2.14E-01
MLCP (cat)	1.33524	5.24E-05	1.03902	5.15E-01	1.4029	3E-05	1.17767	3.06E-02
mTOR	1.11145	2.94E-02	1.02306	6.20E-01	-1.07295	2E-01	1.0235	6.80E-01
NFKBIA	1.32668	8.11E-05	1.04623	4.49E-01	1.24116	3E-03	-1.01402	8.48E-01
NIP3	-1.31389	2.77E-04	1.01847	7.75E-01	-1.13289	9E-02	-1.00126	9.87E-01
NIX	-1.09667	1.57E-01	1.01434	8.23E-01	-1.27477	2E-03	-1.10381	2.14E-01
SPP1	1.1723	1.51E-02	1.03144	6.13E-01	2.87571	9E-15	1.69854	2.80E-08
PDGF-B	1.22638	3.54E-02	1.04626	6.24E-01	1.12241	3E-01	-1.0491	6.71E-01
PGK1	-1.16877	5.44E-04	1.02466	5.34E-01	1.15378	3E-03	1.07181	1.56E-01
PKC-beta1	-1.10665	1.95E-03	-1.08324	1.11E-02	1.05625	1E-01	1.02757	4.49E-01
PSAT	-1.03984	5.18E-01	1.02184	7.20E-01	-1.36842	1E-04	-1.14486	7.64E-02
RhoGDI alpha	-1.07443	1.53E-01	1.00665	8.93E-01	1.20011	3E-03	1.04377	4.79E-01
RUNX2	-1.10966	1.92E-02	-1.06441	1.44E-01	1.25754	6E-05	1.0809	1.37E-01
RXRA	-1.05534	1.58E-01	1.03585	3.49E-01	-1.16056	2E-03	-1.01973	6.69E-01
SERPINA3 (ACT)	3.34376	3.12E-20	1.41743	2.85E-09	1.25436	1E-05	1.22498	1.66E-04
SOCS2	1.26078	1.62E-04	1.02139	6.83E-01	1.82416	1E-08	1.07687	3.30E-01
SOCS3	1.34572	8.59E-05	1.03211	6.15E-01	1.53187	3E-06	1.31016	1.73E-03
STAT3	1.07983	2.97E-01	1.05883	4.35E-01	1.49375	5E-05	1.08846	3.46E-01
Thrombospondin 1	-1.30903	1.04E-02	-1.19903	7.24E-02	1.90846	5E-06	1.44564	4.89E-03
VCAM1	-1.07708	3.31E-01	-1.0911	2.56E-01	1.23678	2E-02	1.11187	2.59E-01

Suppl. Table 2

Unique genes from top 15 pathways in the brown module with relative fold change and p-values in each pair-wise comparison ranked in alphabetical order.

SUPPLEMENTARY

Suppl. Table 3

Transcription Factors									
**IDs in active data set**	**Object name**	**Actual**	**n**	**R**	**N**	**Expected**	**Ratio**	**p-value**	**z-score**
	CREB1	278	828	5126	38450	110.4	2.518	1.73E-51	17.32
**MYC**	c-Myc	174	828	2522	38450	54.31	3.204	1.12E-43	16.99
	SP1	110	828	1648	38450	35.49	3.1	5.22E-26	12.92
	p53	100	828	1283	38450	27.63	3.619	8.06E-29	14.16
	ESR1 (nuclear)	99	828	1289	38450	27.76	3.567	4.91E-28	13.9
	CUX1 (p110)	84	828	1398	38450	30.11	2.79	3.30E-17	10.12
	Androgen receptor	76	828	1013	38450	21.81	3.484	4.73E-21	11.89
	Oct-3/4	74	828	1243	38450	26.77	2.765	4.63E-15	9.382
	RelA (p65 NF-kB subunit)	66	828	939	38450	20.22	3.264	5.55E-17	10.42
	c-Jun	65	828	835	38450	17.98	3.615	5.96E-19	11.33
	SOX2	63	828	630	38450	13.57	4.644	5.24E-24	13.68
	E2F1	62	828	855	38450	18.41	3.367	1.19E-16	10.39
**STAT3**	STAT3	60	828	708	38450	15.25	3.935	2.47E-19	11.7
	NANOG	59	828	732	38450	15.76	3.743	5.29E-18	11.12
	HIF1A	59	828	726	38450	15.63	3.774	3.60E-18	11.19
	YY1	55	828	1148	38450	24.72	2.225	3.95E-08	6.25
	ZNF143	52	828	988	38450	21.28	2.444	4.49E-09	6.822
	C/EBPbeta	48	828	643	38450	13.85	3.467	1.26E-13	9.357
	GCR	44	828	488	38450	10.51	4.187	1.83E-15	10.51
	GATA-3	41	828	647	38450	13.93	2.943	1.14E-09	7.393
	EGR1	38	828	454	38450	9.777	3.887	1.53E-12	9.179
	STAT1	34	828	361	38450	7.774	4.374	8.70E-13	9.554
	NF-kB1 (p50)	34	828	473	38450	10.19	3.338	1.29E-09	7.59
	SRF	34	828	413	38450	8.894	3.823	3.62E-11	8.557
	SP3	34	828	521	38450	11.22	3.03	1.46E-08	6.922
	ETS1	33	828	395	38450	8.506	3.88	4.77E-11	8.534
	E2F4	33	828	343	38450	7.386	4.468	1.05E-12	9.57
	HNF4-alpha	33	828	525	38450	11.31	2.919	5.76E-08	6.568
	C/EBPalpha	32	828	402	38450	8.657	3.696	3.19E-10	8.063
	TCF7L2 (TCF4)	32	828	383	38450	8.248	3.88	9.34E-11	8.403
	PPAR-gamma	32	828	322	38450	6.934	4.615	9.64E-13	9.664
	KLF4	31	828	328	38450	7.063	4.389	8.08E-12	9.144
**FOS**	c-Fos	31	828	361	38450	7.774	3.988	9.26E-11	8.461
	p63	30	828	445	38450	9.583	3.131	5.03E-08	6.707
	SMAD3	29	828	268	38450	5.771	5.025	1.35E-12	9.809
	IRF4	26	828	434	38450	9.346	2.782	3.47E-06	5.539

Suppl. Table 3

Key transcription factors identified for the brown module.

Actual: number of network objects in the activated dataset(s) which interact with the chosen object

n: number of network objects in the activated dataset(s)

R: number of network objects in the complete database or background list which interact with the chosen object

N: total number of gene-based objects in the complete database or background list

Expected: mean value for hypergeometric distribution (n*R/N)

Ratio: connectivity ratio (Actual/Expected)

P-value: probability to have the given value of Actual or higher (or lower for negative z-score)

Z-score: z-score ((Actual-Expected)/sqrt(variance))

SUPPLEMENTARY

Suppl. Fig. 1

A

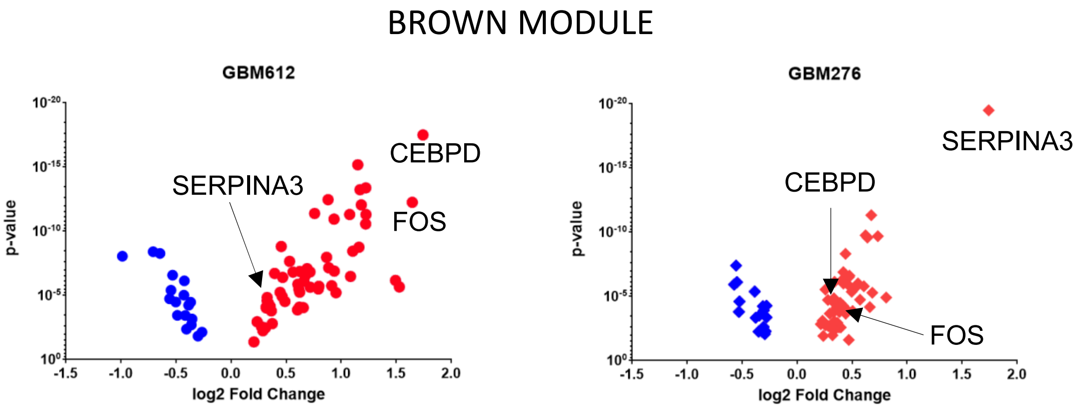

B

Enrichment by Process Networks - Brown Module					
**Networks**	**Total genes**	**p-value**	**FDR**	**DEGS in Data**	**Network Objects from Active Data**
Cell adhesion_Cell-matrix interactions	135	1.574E-04	1.527E-02	7	CD44, ITGA3, Thrombospondin 1, BETA-IG-H3, CD44 soluble, CD44 (ICD), CD44 (EXT)
Proliferation_Positive regulation cell proliferation	150	1.906E-03	6.164E-02	6	MLCP (cat), ZFP36L2, Bcl-6, N-Myc, Neuropilin-1, c-Fos
Inflammation_IL-6 signaling	75	2.846E-02	3.752E-01	3	SERPINA3 (ACT), SOCS3, c-Fos
Inflammation_Interferon signaling	79	3.253E-02	3.752E-01	3	IL10RB, SOCS3, c-Fos
Cell adhesion_Integrin-mediated cell-matrix adhesion	140	3.482E-02	3.752E-01	4	ITGA3, Flotillin-1, SPP1, Tubulin beta 2

C

Enrichment by GO Processes - Brown Module					
**Processes**	**Total genes**	**p-value**	**FDR**	**DEGS in Data**	**Network Objects from Active Data**
regulation of developmental process	2217	4.537E-07	1.188E-03	33	ZFP36L2, Tissue factor, CD44, PQBP-1, ITGA3, TNF-R1, CNTN1 (F3), Bcl-6, Thrombospondin 1, TEF-4, DMP4, S100A13, C6orf134, TESC, NELL1, Flotillin-1, SPP1, BAMBI, NRCAM, ZFP36(Tristetraprolin), WARS, FZD5, DDHD2, N-Myc, Semaphorin 4B, SOCS3, LAGY, ZNF261, Neuropilin-1, Ephrin-B receptor 1, Phox1 (PRRX1), c-Fos, C/EBPdelta
regulation of cell differentiation	1545	4.475E-06	3.853E-03	25	ZFP36L2, CD44, PQBP-1, ITGA3, TNF-R1, CNTN1 (F3), Bcl-6, TEF-4, DMP4, C6orf134, TESC, NELL1, Flotillin-1, SPP1, BAMBI, NRCAM, ZFP36(Tristetraprolin), N-Myc, Semaphorin 4B, SOCS3, LAGY, Neuropilin-1, Phox1 (PRRX1), c-Fos, C/EBPdelta
blood vessel development	467	5.595E-06	3.853E-03	13	CD44, Thrombospondin 1, TEF-4, HOXA13, BETA-IG-H3, NRCAM, WARS, FZD5, PLC-delta 3, SOCS3, Neuropilin-1, Ephrin-B receptor 1, Phox1 (PRRX1)
regulation of multicellular organismal development	1717	9.130E-06	4.190E-03	26	ZFP36L2, Tissue factor, CD44, PQBP-1, ITGA3, TNF-R1, CNTN1 (F3), Bcl-6, Thrombospondin 1, DMP4, TESC, NELL1, Flotillin-1, SPP1, BAMBI, NRCAM, ZFP36(Tristetraprolin), WARS, FZD5, N-Myc, Semaphorin 4B, LAGY, Neuropilin-1, Ephrin-B receptor 1, Phox1 (PRRX1), c-Fos
vasculature development	491	9.599E-06	4.190E-03	13	CD44, Thrombospondin 1, TEF-4, HOXA13, BETA-IG-H3, NRCAM, WARS, FZD5, PLC-delta 3, SOCS3, Neuropilin-1, Ephrin-B receptor 1, Phox1 (PRRX1)

Suppl. Fig. 1. Brown module analysis

A. Volcano plot showing the expression levels of DEGs derived from the 2 cell lines analyzed in the brown module. GBM612 is represented by circles, GBM276 is represented by diamonds. **B.** Enrichment analysis by process networks in common DEGs in the brown module. Terms are ranked by p-value. **C.** Enrichment by GO processes in common DEGs in the brown module. Terms are ranked by p-value.

SUPPLEMENTARY

Suppl. Fig. 2

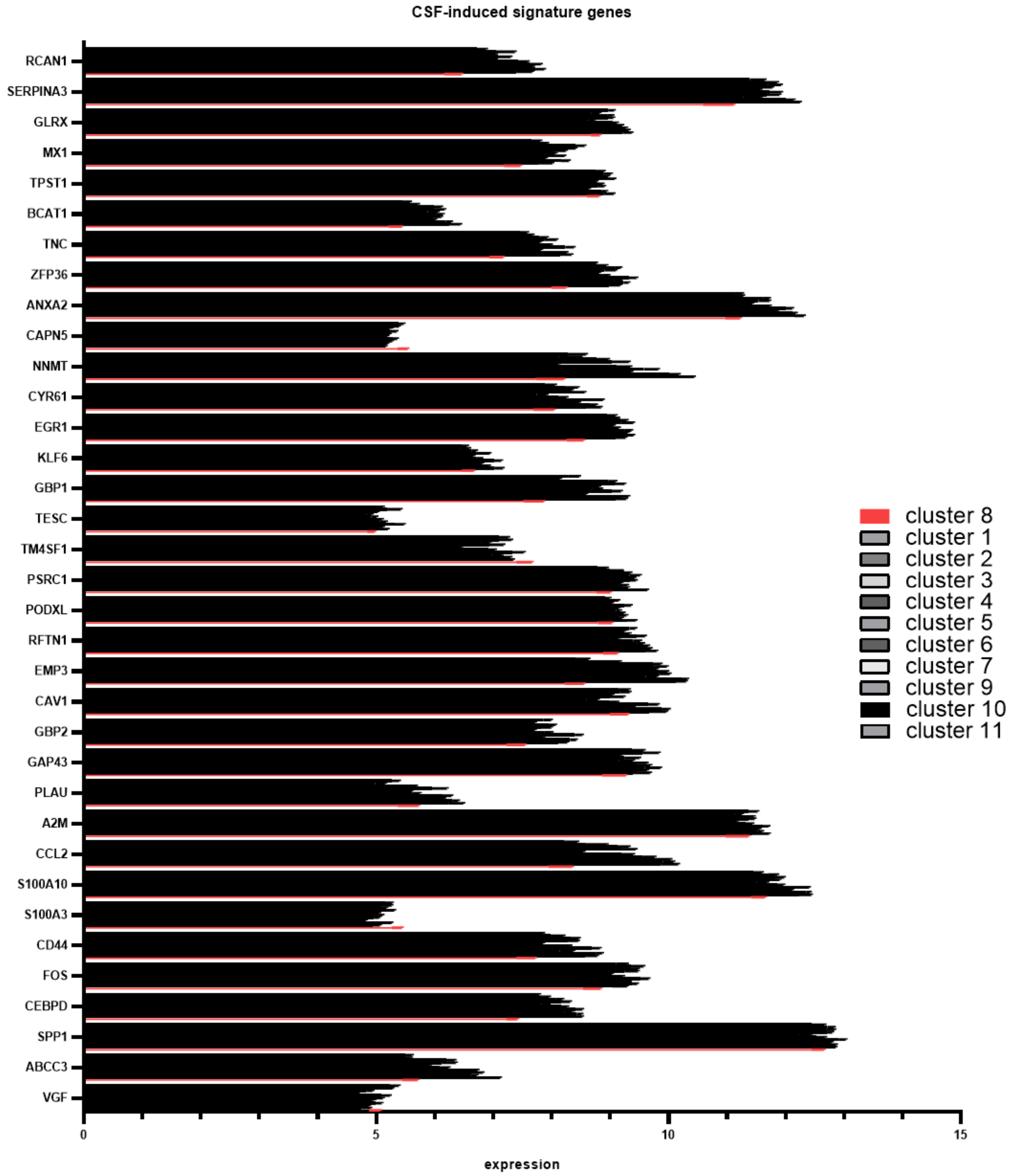

Suppl. Fig. 2 **CSF-induced signature genes expression in each patient cluster**

Plot shows expression average in each cluster for all the top 35 CSF-upregulated genes. Cluster 8 presents a general lower expression for most of the signature genes compared to the other clusters.

SUPPLEMENTARY

Suppl. Fig. 3

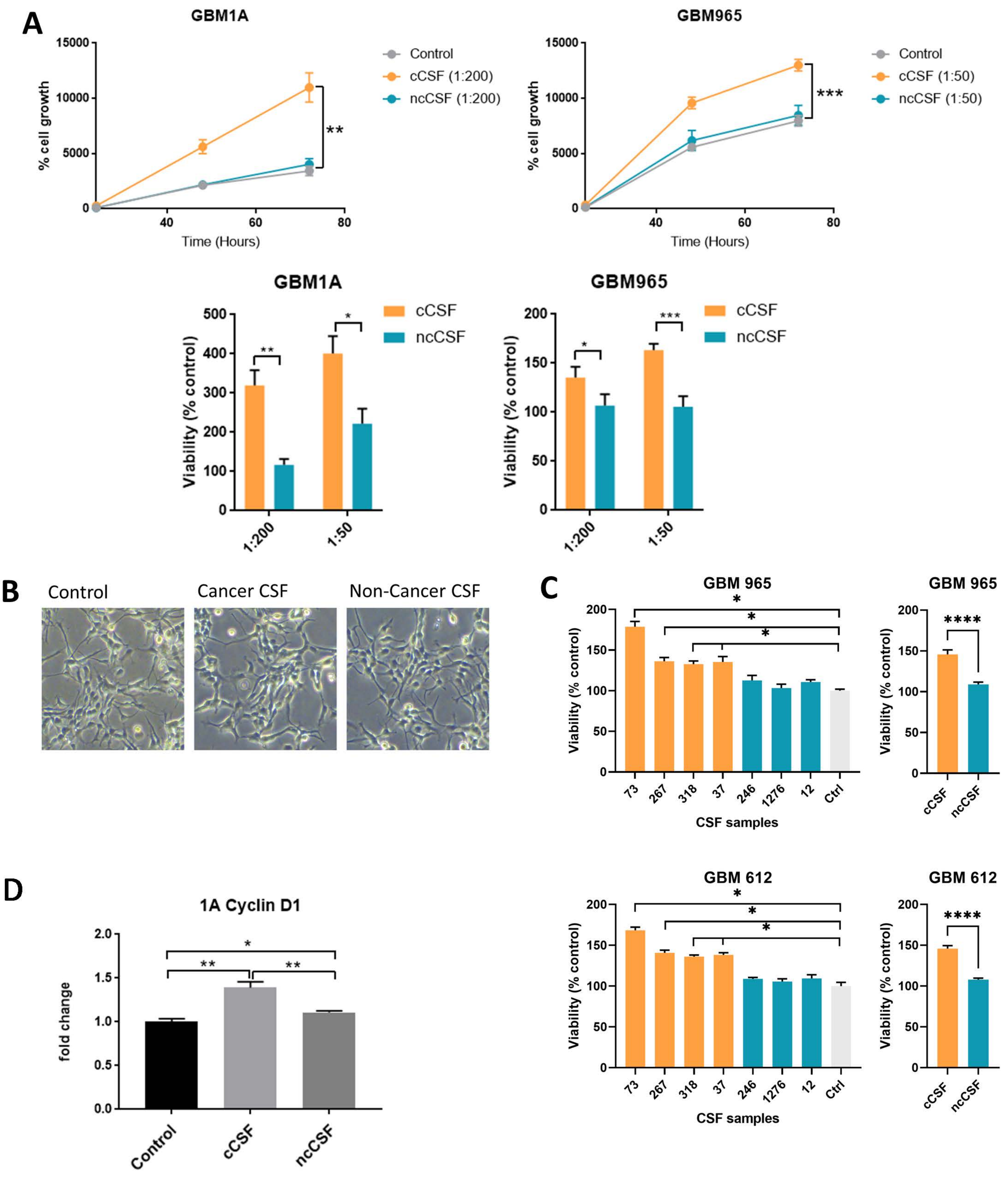

Suppl. Fig. 3 **CSF effects on proliferation**

A. Response to CSF is variable among GBM cells when proliferation is measured. GBM965 responds to CSF at higher concentrations (1:50 compared to 1:200 for GBM1A). (cCSF on GBM1A: 272.13% increase vs ncCSF, p= 0.0081; 319.58% increase vs control, p= 0.0017; cCSF on GBM965: 153.57% increase vs ncCSF, p=0.0045; 163.39% increase vs control, p= 0.0004). **B.** Bright field microscope images at 10X showing no differences in morphology in GBM cells treated with cCSF and ncCSF compared to untreated cells. **C.** CSF samples derived from different patients showed consistently that CSF from non-cancer patients induced moderate response in primary GBM lines as compared to cancer-derived CSF, when viability was used as read-out. Alamar blue assay was run for 72hrs in parallel in 2 GBM lines (GBM965 and GBM612) treated with cCSF (orange) and ncCSF (blue) samples (1:200). **D.** Cyclin D1 expression was measured by qPCR after 24 hours exposure to cCSF or ncCSF in GBM1A cells, revealing a significan increase of Cyclin D1 in cCSF treated cells. 138.92% increase vs untreated, p=0.0021; 125.52% increase vs ncCSF, p=0.0068). Scale bars = mean +/- SEM. * p<0.05, ** p<0.01, ***p<0.001, ****p<0.0001.

SUPPLEMENTARY

Suppl. Fig. 4

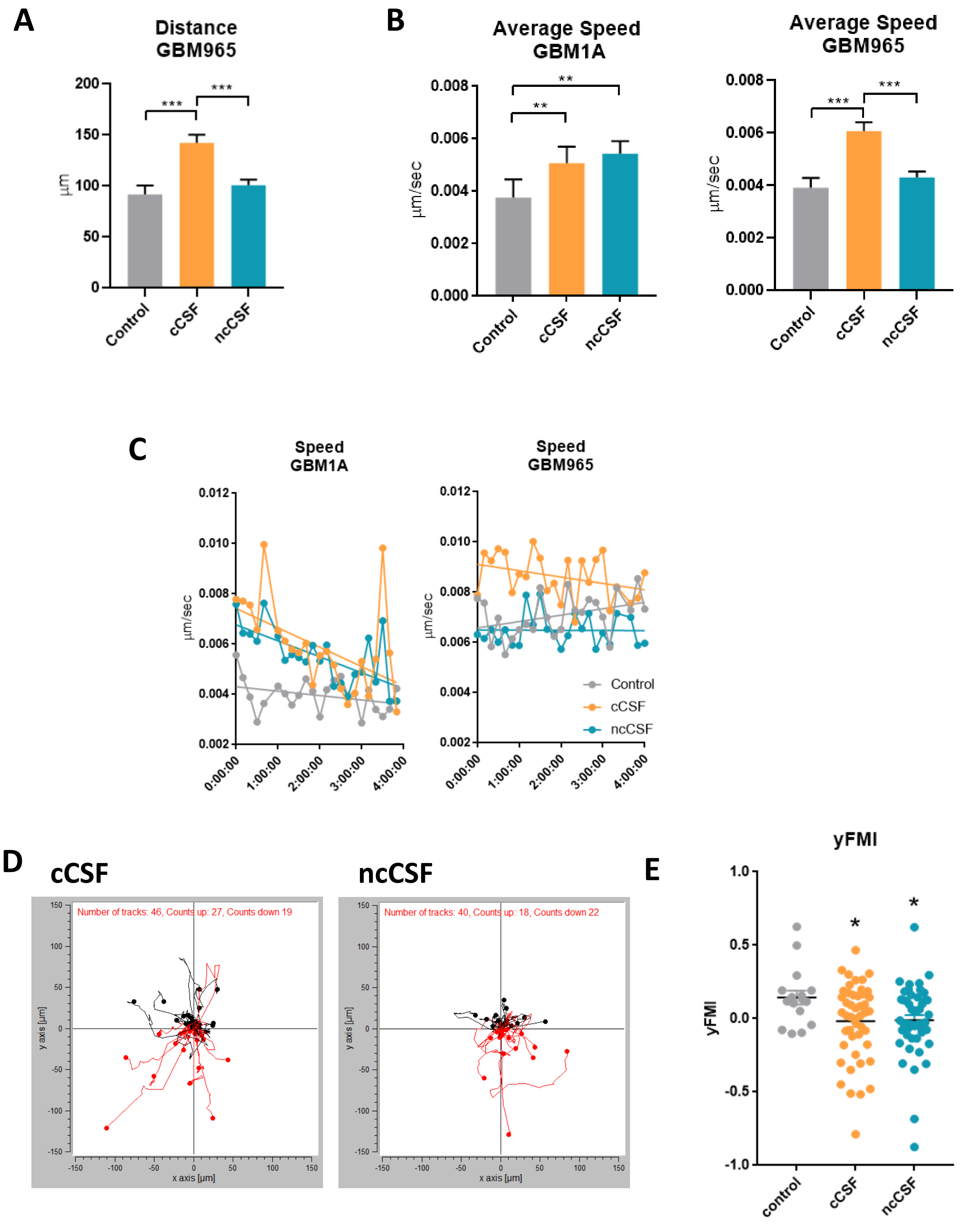

Suppl. Fig. 4 **2D gradient migration assay**

A. Average distance migrated by GBM965 in a CSF gradient shows increased migration in cCSF treated cells. **B.** Shows average speed and **C.** shows speed by frame of GBM cells (GBM1A and GBM965) cultured in a 2D gradient of CSF. Analysis of time-lapse videos acquired over 24 hour period revealed that cells speed is significantly increased during the first 4 hour upon treatment. Although no significant differences in speed were measured between cCSF and ncCSF. **D.** Paths of individual cells analyzed in spider plots showing directionality of movements in the 2D gratient. **E.** Forward migration indices represent the efficiency of forward migration of cells parallel to the gradient (in our assay on the Y axis). CSF shows a chemo-repellant effect on GBM cells migration.

SUPPLEMENTARY

Suppl. Fig. 5

A

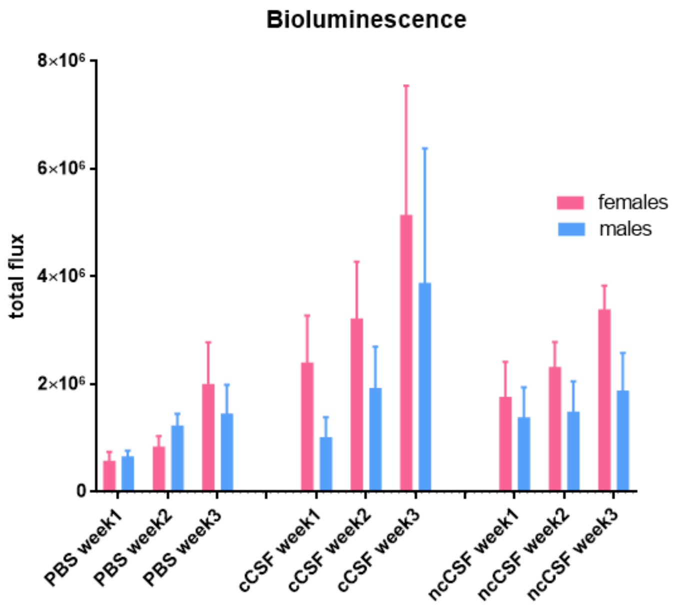

B

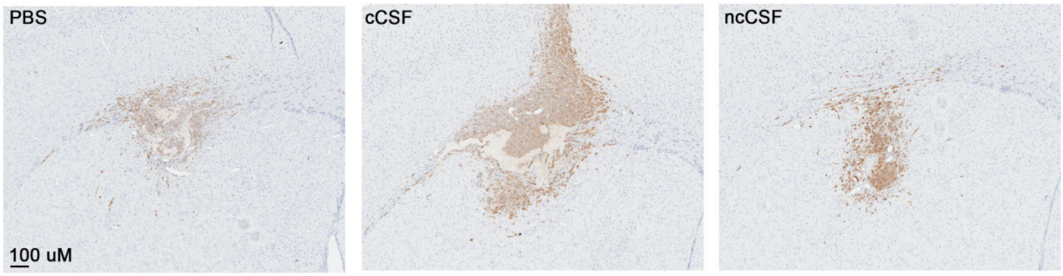

Suppl. Fig. 5 **Tumor growth in vivo**

A. Bioluminescence total flux of mice carrying GBM xenographs showing higher emission in mice co-injected with cCSF as compared to controls and ncCSF injected mice. **B.** Immunohistochemical staining for eGFP (1:1000, A11122, Thermo Scientific) in xenograph derived tissue, showing a larger tumors in the cCSF coinjected cohort.
